# Epidemic Dysentery among the Orphans at Girard College

**Published:** 1852-01

**Authors:** 


### *Epidemic Dysentery among the Orphans at Girard College.*

Dr. Hollingsworth stated that a severe epidemic of diarrhœa and dysentery had recently prevailed among the orphans at the Girard College. It commenced on the fourth day of last month, since which time upward of one hundred cases had occurred; namely, twenty-five of dysentery, and the remainder of diarrhœa with slight dysenteric symptoms. Three of the cases of dysentery terminated fatally, and one death from convulsions occurred in a patient who had passed through the disease.

Few of the patients had much sickness of stomach, nor were any of them affected with tympanitis; their tongues were, in general, clean. In those who recovered, convalescence proceeded very slowly.

All the cases of diarrhœa were treated by oil and laudanum—rest in bed, and a diet of arrow root. Under this treatment all of them recovered.

In the cases of dysentery, the patients were placed, for the first two or three days, on calomel and Dover's powder, followed by sugar of lead and opium. Rochelle salt and laudanum were tried in three cases: at the end of three days, finding the patients getting worse, they were discontinued. Nitrate of silver combined with opium was also given a trial. Depletion and counter-irritation by blisters were ordered in a few instances, but no benefit was derived from the use of either of them. The treatment by calomel and Dover's powder, followed by sugar of lead and opium, was most relied upon, and proved in the end the most satisfactory. Emollient enemeta of starch and laudanum, lard and laudanum, &c., in all instances were ordered during the first stage of the disease. Generally they give but little satisfaction, as they were soon rejected, and often had to be discontinued, from the pain and distress they produced. In the more advanced stages of the complaint, injections of sulphate of zinc and of nitrate of silver were tried in several instances, but with no decided benefit. Arrow root constituted the diet for the first few days. When more support was required, mutton soup, milk punch, &c., were given. Ice was allowed to all.

No local cause could be discovered, to which the origin of the epidemic could be attributed. There is a pond of fresh water within two squares of the College buildings, where the boys are accustomed to bathe. The sleeping apartments are spacious, and most of them well ventilated; the temperature of the interior of the main building is considerably lower than that of the atmosphere out of doors, but to none of these causes was it thought, could the extensive prevalence of the disease be attributed.

Two of the domestics employed in the college were attacked with dysentery. One of them was removed to-day to St. Joseph's Hospital. The wife of the president has also been taken sick.*

With the view of arresting the epidemic, some two hundred and fifty of the orphans have been sent a few days in advance of their expected vacation to the homes of their relatives. The epidemic has also made its appearance at the Orphan Asylum, situated upwards of a mile south of the College.

To Drs. Pepper and Page, Dr. H. stated that his colleague and himself were much indebted for valuable advice and assistance rendered on several occasions during the prevalence of the epidemic.

Dr. Condie did not think that it was to local causes alone that we were to look for the production of the dysentery among the orphans at Girard

* Dr. H. regrets to state that this case has terminated fatally. The lady left Philadelphia the morning succeeding the night of her attack, on a visit to some members of her family at Bellefont, near which place, after ten or twelve days' severe illness, she expired. The domestic mentioned as being removed to St. Joseph's Hospital, also died. She was sent there immediately on being taken sick.

College. There was evidently a general morbid condition of the atmosphere predisposing to that disease. He had met with it very frequently, in both children and adults, during the last two months. It prevails, certainly, to a very great extent in the Southern districts. On referring to the returns made to the Board of Health of the interments of the city and liberties, he found that, during the month of June, fifty deaths were reported from dysentery, and during the month of July, ninety-eight; total, 148. In the cases that have fallen under his notice, the disease has varied very much in the severity of its symptoms. In many it was very slight, and gave way readily to the simplest treatment, whilst, in others, it was very severe and obstinate, and occasionally proved fatal, even under the most vigorous treatment, commenced at the very onset of the attack. The disease was, in nearly all cases, attended with fever. In some, however, the fever was very slight, and of short duration; in others, it was more intense, and continued until the termination of the attack. In a number of cases, it assumed the characteristics of remittent fever, with two exacerbations in the course of the day. The tormina and tenesmus were, in many cases, peculiarly severe and almost continuous. The pulse varied very much in different cases; it was mostly quick and frequent, and small in volume, and often tense. Occasionally, however, it was somewhat full and soft; seldom slow and languid, excepting in cases where, by the long continuance and violence of the attack, the strength of the patient had become considerably exhausted. The tongue, in many cases, was clean and red; in some, however, it was coated, especially along its centre, with a dirty white, or yellowish fur. The stomach was, in the greater number of cases, very irritable. Nausea was generally present, while spontaneous vomiting of a fluid colored with bile was not unfrequent. More commonly, however, the vomiting only occurred upon medicine or drinks being taken into the stomach. Many patients complained of a soreness, some of actual pain of the abdomen. Tenderness of this part was very commonly indicated upon slight pressure being applied. The discharges from the bowels were very various. In some cases, after severe and prolonged straining, a small quantity of mucus, more or less stained with blood, was passed. In other cases, the discharge was of a jelly-like consistence, either entirely colorless, mixed with blood, or of a dark slate color. The discharges were often frothy. Pure blood, with a very slight admixture of mucus, was avoided in some cases, and occasionally the hemorrhagic stools were frequent and profuse. The cases in which these latter occurred were always protracted and unmanageable, often fatal. The stools had, in general, a peculiar odor, more marked in some cases than in others. The disease, when of any severity, run, in general, a protracted course. Convalescence was, also, slow and tedious.

In regard to treatment, Dr. Condie remarked that in no case had he administered, either in the commencement or during the continuance of the disease, the usual prescription of castor oil, with or without the addition of laudanum. He believed that all purgatives, even the mildest, were calculated to increase the tormina and tenesmus, by the irritation they produced in the inflamed coats of the intestines, while he knew of no urgent indication they were calculated to fulfill. He was equally averse from the employment, in dysentery, of injections. He has seldom seen them produce, even when of an anodyne and emollient nature, more than temporary relief. These views in relation to the employment of purgatives by the mouth, and injections into the rectum, in cases of dysentery, he was happy to find adopted by many of the best practitioners of Europe. He believed that by far the most rational and effective plan of treatment in dysentery was to arrest the abnormal action of the intestines; "to put them in splints," to use an expressive phrase of the late Dr. Parrish, by the administration of opiates and astringents. Dr. Condie had been much pleased by the effects of the nitrate of silver in dysentery. He gave it in the dose of one-fourth of a grain, combined with from one-third to one-half of a grain of opium, every three hours to an adult, during the day, and at night a pill composed of from one to one grain and a half of opium, one grain of ipecacuanha, and five grains of the extract of gentian. To children over four years of age, he has given one-sixth of a grain of the nitrate of silver, and the same quantity of opium, every three hours, with three grains of Dover's powder at night. In those cases attended by bloody discharges, he prefers the acetate of lead to the nitrate of silver, in doses of one grain, combined with from a third to one half a grain of opium every three hours, for an adult, and with a proportionally smaller amount of opium for a child. Dr. Condie was formerly of the opinion that opium could not be administered with safety in the earlier stages of dysentery: experience has convinced him, however, that the earlier it is resorted to, in doses adapted to quell the tormina and tenesmus, the quicker will the disease be cut short.

Perfect rest in a recumbent position, is all important in every case of dysentery; the more, also, the patient resists the calls to stool, the better; children especially should not be allowed to rise every time they desire, nor should they be allowed to sit for a long period, ineffectually straining to obtain an evacuation from their bowels.

Warm fomentation to the abdomen with a strong decoction of hops, or soft emollient poultices to this part, have appeared to him to give great ease to the patient. To do good, however, they must be properly applied, and continued for a length of time without intermission.

Bleeding from the arm he has found beneficial in many cases. In cases occurring in robust adults, with intense tormina and tenesmus, decided febrile reaction, a quick, frequent, and tense pulse, he has known the loss of a moderate quantity of blood to be followed by prompt and decided relief. Where there has been much soreness or tenderness of the abdomen, cups to this part he has often found beneficial. Blisters to the abdomen Dr. C has occasionally resorted to, and in a few cases they have appeared to act beneficially. He cannot say, however, that their good effects, in the generality of cases, were of so decided a character as to compensate for the discomfort invariably produced by their employment.

When the tenesmus and tormina have considerably abated, and the discharges have become less mixed with blood, Dr. Condie has been accustomed to administer, at night, five grains of the blue mass and one grain of opium, which he has generally found to occasion dark consistent discharges, soon followed by natural stools.

During the height of the disease, there is seldom any desire for food, nor should any be allowed. Cold water, or cold toast water, slightly acidulated with lemon juice, may be given to allay thirst. Fresh buttermilk is often agreeable to the patient, and he may be allowed to partake of it in moderation. —*Quarterly Summary of Transactions of College of Physicians of Phil.*

